# A cohort analysis of patients receiving neoadjuvant androgen deprivation therapy prior to robot-assisted laparoscopic prostatectomy during the Covid-19 pandemic

**DOI:** 10.1177/20514158211022216

**Published:** 2021-06-07

**Authors:** Sahan S Bennett, Hing Y Leung, Imran Ahmad

**Affiliations:** 1 College of Medical, Veterinary and Life Sciences, University of Glasgow, G12 8QQ, United Kingdom; 2 The Beatson Institute for Cancer Research, Glasgow, G61 1BD, United Kingdom; 3 Institute of Cancer Sciences, University of Glasgow, United Kingdom

**Keywords:** Prostate cancer, localised, neoadjuvant, androgen deprivation therapy, Covid-19

## Abstract

**Objectives:**

The purpose of this study was to investigate localised prostate cancer treated with or without neoadjuvant androgen deprivation therapy prior to robot-assisted laparoscopic prostatectomy, and the impact of Covid-19 treatment disruption, on clinico-pathologic outcomes.

**Patients and methods:**

Data was retrospectively collected from 124 consecutive patients treated with robot-assisted laparoscopic prostatectomy between November 2019–September 2020. Sixty-two patients were treated before 13 March 2020 (historic cohort) and 62 afterwards (covid cohort). Thirty-seven patients in the covid cohort additionally received neoadjuvant androgen deprivation therapy (mean duration of 3 months) consisting of bicalutamide 150 mg once a day for 4 weeks, with leuprolide 3.75 mg monthly injections commencing after week 1, up until the date of surgery.

**Results:**

Statistical analysis found no difference in peri-operative measures and length of stay for patients treated with or without neoadjuvant androgen deprivation therapy. Patients with delayed surgical treatment offered neoadjuvant androgen deprivation therapy showed a trend towards a reduction in positive surgical margins (*p*=0.134), N1 disease (*p*=0.424) and pathological down-staging (50% patients with pT2 disease). Patients within the covid cohort experienced significantly increased detectable prostate-specific antigen levels (*p*<0.007).

**Conclusion:**

Our study demonstrated that a three-month duration of neoadjuvant androgen deprivation therapy prior to robot-assisted laparoscopic prostatectomy may improve pathological outcomes but this time-frame is inadequate to influence detectable prostate-specific antigen levels. Covid-19-related treatment delays led to significantly increased detectable prostate-specific antigen levels.

**Level of evidence:**

2b

## Introduction

Amidst the current Covid-19 pandemic, prostate cancer (PCa) patients have experienced postponements to their curative surgical treatments on a global scale.^1^ One of the most significant effects due to delay in surgery is psychological morbidity.^2,3^ Little is known about the oncological outcomes, particularly in a contemporary cohort of high-risk locally-advanced prostate cancers. The current management strategies of PCa patients are mostly driven by findings from studies conducted prior to the Covid-19 pandemic. Many centres have delayed their prostatectomy treatment, whilst a few centres have opted to continue performing surgeries during this time. To address the delay, some institutions have trialled neoadjuvant androgen deprivation therapy (neoADT) prior to robot-assisted laparoscopic prostatectomy (RALP).^4,5^ whilst others have opted for watchful waiting.^3^ Trialling neoADT is not surprising as the treatment modality of choice, given that previous studies assessing neoADT have shown promise through reduction of post-surgical margins, but without any benefit on long-term patient and surgical outcomes.^6,7^

At the present, there is a lack of a strong evidence base for management of PCa patients during Covid-19 as informed by the analysis of patient outcomes during the initial months of the pandemic itself. We investigated the impact of the Covid-19 pandemic on patients undergoing radical surgical treatment at our practice and examined whether the delay negatively impacted on surgical and patient outcomes. In particular, our primary objective was to compare the clinic-pathologic outcomes of localised PCa treated with or without neoADT prior to RALP.

## Materials and methods

### Patient selection and study design

One hundred and twenty-four consecutive men, diagnosed with localised PCa and treated with RALP, were selected from our regional centre, the Queen Elizabeth University Hospital (QEUH), Glasgow, UK. Patients were divided into two cohorts, and 62/124 men treated prior to Covid-19 (November 2019–March 2020) formed the historic cohort. The remaining 62/124 patients treated during Covid-19 (March 2020–September 2020) formed the covid cohort. Written informed consent was obtained from patients for their anonymised information to be published in this article. Appropriate ethical approval was obtained as part of the National Health Service (NHS) Greater Glasgow and Clyde (GG&C) Audit programme and patient-related data were securely kept in accordance with the Data Protection Act 1998 and local NHS Trust policy.

### Treatment and follow-up

All 62 men from the historic cohort and 25 patients from the covid cohort received RALP alone. The remaining 37 patients in the covid cohort received neoADT prior to RALP. More specifically, neoADT was initiated with 4 weeks of bicalutamide 150 mg once a day (OD), an anti-androgen. After week 1, all patients transitioned to leuprolide 3.75 mg monthly subcutaneous injections, a luteinising hormone release hormone agonist (LHRHa), with bicalutamide continuing on for a further 3 weeks alongside. NeoADT continued up until the surgical date. NeoADT duration was 3 months (mean of 89 days).

All patients were followed up until 6 weeks after RALP, with assessment of serum prostate-specific antigen (PSA) level, acting as our primary endpoint. PSA measurements **⩾**0.1 ng/ml were defined as ‘detectable PSA’.

### Data collection

Patients’ medical records were reviewed to extract clinical, surgical, pathological and biochemical data until September 2020, in a retrospective manner.

We abstracted patient-level variables including age, body mass index (BMI), duration of waiting list occupancy and surgical school attendance. Disease-related parameters included clinical and pathological tumour stage (cT and pT, respectively), post-operative surgical margin (PSM) status, lymph node (LN) status, biopsy Gleason grade and PSA level, measured pre-and-post-operatively. cT and pT were determined according to the seventh edition of the TNM Classification of Malignant Tumours.^8^ Tumour grade of the diagnostic prostate biopsy material and surgical specimens was determined according to Gleason scoring system 2014 guidelines by the International Society of Urological Pathology.^9^

### Statistical analysis

Initial patient characteristics including age, BMI, cT stage, biopsy Gleason grade, pre-operative PSA level acted as co-variables, whilst neoADT provision or cohort status, accounting for treatment delay, acted as response variables. Differences in continuous and categorical variables were examined using the independent-samples *t* test and Chi-square test, respectively. Mean, median, range and standard deviations were generated for continuous variables, and frequencies and proportions were generated for categorical variables.

All data analysis was performed using Statistical Package for the Social Sciences (SPSS) Version 27. All *p* values were two-sided, with the significance level defined as *p*<0.05.

## Results

### Baseline characteristics

Clinic-pathologic characteristics of historic and covid cohorts were comparable (Table 1) with similar age, cT stage, tumour grade (Gleason score) and presenting serum PSA levels.

**Table 1.** Baseline characteristics of patients within the covid and historic cohorts.

Variable	Covid cohort (*n*=62)	Historic cohort (*n*=62)	*p* Value
**Patient characteristics:**			
Mean age (years)	62.79 (SD 6.01)	64.50 (SD 6.38)	0.127
Mean BMI	27.81 (SD 3.64)	27.60 (SD 3.89)	0.757
**Clinical disease characteristics:**			
Mean Biopsy Gleason Group	2.30 (SD 0.92)	2.35 (SD 0.93)	0.720
Mean pre-operative PSA (ng/ml)	10.72 (SD 7.62)	9.09 (SD 4.12)	0.140
**cT stage:** ^a^ **count (%)**			
1	2 (4.0%)	6 (9.7%)	0.837
2	24 (48.0%)	47 (75.8%)	0.161
3a	20 (40.0%)	9 (14.5%)	0.161
3b	3 (6.0%)	0 (0%)	0.406
4	1 (2.0%)	0 (0%)	0.876
**Mean duration of waiting list occupancy (weeks)**	16.34 (SD 7.74)	5.03 (SD 2.41)	<0.0001^b^

BMI: body mass index; cT: clinical T stage; PSA: prostate-specific antigen; SD: standard deviation.

SDs are shown within brackets. Proportions (in %) are shown in brackets.

a cT stage: results from 12 patients within the covid cohort have been omitted from this table due to the unavailability of corresponding pathological T stage results.

b Results with statistical significance.

Among patients within the covid cohort, patients who received neoADT had a significantly higher tumour grade (median of 2.0 and a range of 3) (*p*=0.016). Similarly, the patients receiving neoADT within the covid cohort, had a higher cT stage, with 0% of patients with cT1 disease compared to 12.5% in RALP alone group (*p*=0.036) and with an increased likelihood of high-risk disease according to the Di Amico Classification (83.8% vs 70.8*%, p*=0.230) (Table 2).

**Table 2.** Baseline characteristics of patients within the covid cohort, who received neoadjuvant androgen deprivation therapy (neoADT) prior to robot-assisted laparoscopic prostatectomy (RALP) or RALP treatment alone.

Covid Cohort			
Variable	NeoADT + RALP (*n*=37)	RALP alone (*n*=25)	*p* Value
**Patient characteristics:**			
Mean age (years)	62.16 (SD 6.43)	63.72 (SD 5.48)	0.321
Mean BMI	27.68 (SD 3.90)	28.00 (SD 3.30)	0.734
**Clinical disease characteristics:**			
Mean Biopsy Gleason Group	2.53 (SD 0.85)	1.96 (SD 0.94)	0.016^a^
Mean pre-operative PSA (ng/ml)	9.89 (SD 7.77)	11.95 (SD 7.37)	0.300
**cT stage:** ^b^ **count (%)**			
1	0 (0%)	2 (12.5%)	0.036^a^
2	16 (47.1%)	8 (50%)	0.841
3a	15 (44.1%)	5 (31.3%)	0.368
3b	2 (5.9%)	1 (6.3%)	0.920
4	1 (2.9%)	0 (0%)	0.484
**Di Amico Risk Group:** ^c^ **count (%)**			
High	31 (83.8%)	17 (70.8%)	0.230
Intermediate	5 (13.5%)	4 (16.7%)	0.764
Low	1 (2.7%)	3 (12.5%)	0.134
**Mean duration of waiting list occupancy (weeks)**	15.67 (SD 6.96)	17.34 (SD 8.81)	0.410

BMI: body mass index; cT: clinical T stage; PSA: prostate-specific antigen; SD: standard deviation.

SDs are shown within brackets. Proportions (in %) are shown in brackets.

a Results with statistical significance.

b cT stage: results from three patients in the neoADT group and nine patients from the RALP alone group have been omitted from this table due to the unavailability of corresponding pathological T stage results.

c Di Amico Risk Group: one patient in the RALP alone group was not included in the table due to the unavailability of the clinical T stage result.

The only difference amongst baseline characteristics between the groups was the length of wait till surgery. Compared to the historic cohort, the covid cohort patients experienced a significantly lengthier wait till surgery (16.34 weeks vs 5.03; *p*<0.0001), shown in Table 2.

### Peri-operative and immediate post-operative period

Wait till surgery and neoADT status had no impact on peri-operative and immediate post-operative measures, in terms of volume of blood loss, operating times and length of hospital stay (LOS) (Table 3). All groups within both cohorts recorded a median LOS of 3 days. Within the covid cohort, there is a trend of neoADT in reducing operation times compared to RALP alone therapy (135.57 min vs 165.72 min, *p*=0.814) (Table 3).

**Table 3.** Comparison of peri-operative and immediate post-operative parameters amongst patients within the covid and historic cohorts.

	Covid cohort			Both cohorts		
Variable	NeoADT + RALP (*n*=37)	RALP alone (*n*=25)	*p* Value	Covid cohort (*n*=62)	Historic cohort: RALP alone (*n*=62)	*p* Value
**Mean operation time (min)**	137.57 (SD 58.56)	165.72 (SD 58.85)	0.814	146.78 (SD 59.62)	143.81 (SD 47.26)	0.764
**Mean blood loss (ml)**	348.65 (SD 241.38)	308.33 (SD 170.85)	0.327	335.45 (SD 219.99)	295.16 (SD 206.197)	0.309
**Length of hospital stay:**						
Mean	2.60	2.71	0.753	2.63	2.65	0.968
SD	1.48	0.92		1.31	1.52	
Median	3.00	3.00		3.00	3.00	

NeoADT: neoadjuvant androgen deprivation therapy; RALP: robot-assisted laparoscopic prostatectomy; SD: standard deviation.

SDs are shown within brackets.

### Pathological indicators of prognosis at the time of RALP

Pathological findings, including PSMs, pT-stage and LN status (pN), were compared amongst both cohorts (Table 4).

**Table 4.** Comparison of pathological indicators of prognosis amongst patients within the covid and historic cohorts.

	Covid cohort			Both cohorts		
Variable	NeoADT + RALP (*n*=37)	RALP alone (*n*=25)	*p* Value	Covid cohort (*n*=62)	Historic cohort: RALP alone (*n*=62)	*p* Value
**Surgical margins** ^a^						
Positive	6 (17.6%)	6 (37.5%)	0.134	12 (24.0%)	20 (32.3%)	0.317
Negative	28 (82.4%)	10 (62.5%)	0.134	38 (76.0%)	42 (67.7%)	0.317
Margin location:						
**Apical:** Count (%)						
Negative	31 (91.2%)	13 (81.3%)	0.317	44 (88.0%)	52 (83.9%)	0.835
<3 mm	2 (5.9%)	3 (18.8%)	0.162	5 (10.0%)	8 (12.9%)	0.882
⩾3 mm	1 (2. 9%)	0 (0.0%)	0.484	1 (2.0%)	2 (3.2%)	0.923
**Basal:** Count (%)						
Negative	32 (94.1%)	14 (87.5%)	0.424	46 (92.0%)	55 (88.7%)	0.835
<3 mm	1 (2.9%)	0 (0.0%)	0.484	1 (2.0%)	2 (3.2%)	0.923
⩾3 mm	1 (2.9%)	2 (12.5%)	0.194	3 (6.0%)	5 (8.1%)	0.923
**Circumferential:** Count (%)						
Negative	31 (91.2%)	14 (87.5%)	0.689	45 (90.0%)	54 (87.1%)	0.882
<3 mm	2 (5.9%)	2 (12.5%)	0.424	4 (8.0%)	8 (12.9%)	0.726
⩾3 mm	1 (2.9%)	0 (0.0%)	0.484	1 (2.0%)	0 (0%)	0.546
**pT stage**^b^ Count (%)						
2	17 (50.0%)	7 (43.8%)	0.698	24 (48.0%)	35 (56.5%)	0.667
3a	11 (32.4%)	8 (50.0%)	0.230	19 (38.0%)	23 (37.0%)	0.995
3b	6 (17.6%)	1 (6.3%)	0.271	7 (14%)	4 (6.5%)	0.430
**pN status**^c^ Count (%)						
Positive (N1)	5 (20.0%)	3 (33.3%)	0.424	8 (23.5%)	2 (7.7%)	0.110
Negative (N0)	20 (80.0%)	6 (66.7%)	0.424	26 (76.5%)	24 (92.3%)	0.110
**Mean number of assessed nodes per patient**	21.36 (SD 5.48)	19.00 (SD 4.39)	0.254	20.74 (SD 5.25)	19.54 (SD 7.88)	0.507
**Mean number of sampled nodes with cancer**	0.52 (SD 1.33)	0.67 (SD 1.32)	0.778	0.56 (SD 1.31)	0.08 (SD 0.27)	0.043^d^

NeoADT: neoadjuvant androgen deprivation therapy; RALP: robot-assisted laparoscopic prostatectomy; SD: standard deviation.

SDs are shown within brackets. Proportions (in %) are shown in brackets.

a Surgical margins: due to the unavailability of pathological data within the covid cohort, results from three patients in the neoADT group and nine patients from the RALP alone group are not present in the table.

b pT stage: due to the unavailability of pathological data, results from nine patients in covid group are not present in the table.

c pN status: due to unavailability of pathological data in the covid group, results from 12 patients treated with neoADT and 16 patients treated with RALP alone, are not present in the table.

d Results with statistical significance.

For patients within the covid cohort, neoADT demonstrated a trend towards increased negative surgical margins (82.4% vs 62.5%, *p*=0.134) at each margin location: apical (91.2% vs 81.3%), basal (94.1% vs 87.5%) and circumferential (91.2% vs 87.5%). Similarly, within the covid cohort, neoADT demonstrates a trend towards pathological downstaging (50% patients with pT2 disease) compared to RALP alone (50% patients with pT3a disease) and a reduction in positive pN (20% vs 33.3% N1 disease; *p*=0.424).

Our pN results demonstrate a trend of increased N1 disease in the covid cohort compared to the historic cohort (23.5% vs 7.7%, *p*=0.110) coupled with a significantly elevated risk of sampled LNs being positive (*p*=0.043).

### Detectable PSA following RALP

PSA levels, measured 6 weeks after RALP, did not show any statistical difference amongst patients given neoADT or RALP alone, within the covid cohort. Between the covid and historic cohorts, a significantly lower rate of detectable PSA values were found in the historic cohort (3.3% versus 21.7%, *p*=0.007) (Table 5).

**Table 5.** Comparison of post-operative prostate-specific antigen (PSA) levels amongst patients within the covid and historic cohorts.

	Covid cohort			Both cohorts		
Variable	NeoADT + RALP (*n*=37)	RALP alone (*n*=25)	*p* Value	Covid cohort (*n*=62)	Historic cohort: RALP alone (*n*=62)	*p* Value
**Six-week PSA status:**^a^ Count (%)						
Detectable (⩾0.1 ng/ml)	4 (22.2%)	1 (20.0%)	0.920	5 (21.7%)	2 (3.3%)	0.007^b^
Undetectable (<0.1 ng/ml)	14 (77.8%)	4 (80.0%)	0.920	18 (78.3%)	59 (96.7%)	0.007^b^
**Six-week PSA in detectable patients:**						
Mean	0.27	0.20	0.685	0.26	0.45	0.198
Median	0.20	0.20		0.20	0.45	
SD	0.15	0.00		0.13	0.21	

NeoADT: neoadjuvant androgen deprivation therapy; RALP: robot-assisted laparoscopic prostatectomy; SD: standard deviation.

Proportions (in %) are shown in brackets.

a Six-week PSA status: due to the unavailability of data, results from 39 patients in covid cohort and one patient from the historical cohort are not present in the table.

b Results with statistical significance.

### Surgical school attendance and implications

The impact of the Covid-19 pandemic on surgical school parameters were explored in Table 6. There was a significantly higher level of surgical school attendance amongst men treated in the historic cohort compared to the covid cohort (93.5% vs 21%, *p*<0.0001).

**Table 6.** Comparison of surgical school attendance amongst patients within the covid and historic cohorts.

	Covid cohort			Both cohorts		
Variable	NeoADT + RALP (*n*=37)	RALP alone (*n*=25)	*p* Value	Covid cohort (*n*=62)	Historic cohort: RALP alone (*n*=62)	*p* Value
**Surgical school attendance:** Count (%)						
Yes	9 (24.3%)	4 (16.0%)	0.434	13 (21.0%)	58 (93.5%)	<0.0001^a^
No	28 (75.8%)	21 (84.0%)	0.434	49 (79.0%)	4 (6.5%)	<0.0001^a^
**Duration between surgical school and RALP (days):**						
Mean	121.33	146.25	0.201	129.00	16.91	<0.0001^a^
Median	118.00	146.50		119.00	13.00	
SD	22.14	45.88		31.56	13.90	

NeoADT: neoadjuvant androgen deprivation therapy; RALP: robot-assisted laparoscopic prostatectomy; SD: standard deviation.

Proportions (in %) are shown in brackets.

a Results with statistical significance.

## Discussion

### Peri-operative and immediate post-operative period

Our findings remain consistent with the literature, re-enforcing a lack of improvement in peri-operative and immediate post-operative measures after neoADT.^10^ In line with a previous study, we explored this measure using surrogates of blood loss, operative time and LOS.^10^

The trend of neoADT in reducing operation times compared to RALP alone therapy within the covid cohort (135.57 min vs 165.72 min, *p*=0.814) is confounded by inter-surgeon variability, given that at our unit, patients considered for RALP are referred to specific surgeons based on the baseline level of risk. Moreover, patients considered for neoADT at our unit are likely to have a higher baseline risk.

### Pathological indicators of prognosis at the time of RALP

The overwhelming evidence of an improvement of pathological outcomes with neoADT^7,11–13^ is somewhat replicated in our findings.

With a trend of neoADT being prescribed to the more advanced high-risk patients at our unit, often with larger tumour sizes, witnessing increased negative rates of PSM status is very promising, advocating the use of neoADT for PCa patients. However, considerably shrunken tumours^14^ make it trickier to accurately determine the tumour at the inked margins.^15^

The scope for a reduction of nodal metastasis with neoADT amongst the covid cohort patients is explained by the systemic action of androgen deprivation therapy (ADT), readily acting on and transforming micro-metastasis into node-negative (N0) disease,^11^ as well as reducing the potential for metastasis in the first instance.^16^

Initial observations suggest that the prolonged waiting list occupancy due to Covid-19 has not led to worse pathological outcomes in terms of PSMs or pT stage. Our pN findings suggest that it may be likely that the delay prior to RALP, has led to an increased likelihood of disease progression and LN metastasis for patients in the covid cohort.

### Detectable PSA following RALP

Our study did not identify a reduction in detectable PSA levels as a result of neoADT directly, nor with a three-month duration of neoADT. A longer duration of neoADT beyond 3 months,^12,17^ may show more conclusive results. Another study has demonstrated that 8 months duration of neoADT prior to RALP leads to a significant decrease in PSA failure rates.^18^

Our findings of a significant reduction of detectable six-week PSA levels in our historical cohort compared to the covid cohort may be attributable to the Covid-19 treatment delays. This has led to the increased chance of detectable PSA levels amongst patients within the covid cohort. Moreover, the increased likelihood of prostate-confined disease at RALP in the historic cohort, due to shorter waiting times, may explain the significantly reduced detectable PSA levels within this cohort.

### Surgical school attendance and implications

Shortly after an operation is planned, patients are invited to attend a single session of the surgical school. This acts as part of enhanced recovery after surgery (ERAS) scheme, to optimise patient outcomes from their surgery by explanation of what to expect after surgery.^19^ Moreover, patients are primed to be more involved in their care, to increase their levels of motivation to stay fit to meet the physical demands of their operation.

The variation in surgical school attendance rates amongst both cohorts stems from multiple attributable factors linked to the Covid-19 pandemic, including the patients’ fears of Covid-19 infection from hospital attendance and increased levels of isolation amongst cancer patients being more at-risk of poorer outcomes from Covid-19 disease.^1^ Without a postponement to RALP treatment prior to the Covid-19 pandemic, historic cohort patients experienced a significantly shorter timeframe post-surgical school until their operation.

During Covid-19, surgical school has shown promise in terms of increased patient outcomes and healthcare cost savings.^19,20^ Although, in our study, given that LOS and peri-operative measures were no different amongst both cohorts, this makes the true impact of surgical school attendance questionable. Further studies are needed, to confirm our preliminary data and to gauge the long-term patient outcomes, including rates of re-admission. Ideally questionnaire-based studies will be valuable assets to explore patients’ peri-operative experience, depending on their surgical school attendance status.

### Main findings of this study

The provision of neoADT shows a trend of an improvement in pathological outcomes in terms of PSMs, pT and N1 disease. There was no impact on perioperative care and LOS despite a long wait with or without neoADT.

### Limitations

A longer duration of follow-up will enable assessment of whether patients on neoADT have higher rates of biochemical failure after initial undetectable PSA levels. In our institute, RALP was performed by three different surgeons, potentially introducing bias into the study.

## Conclusion

During the Covid-19 pandemic, patients experienced a significantly lengthier waiting list occupancy, higher rates of surgical school non-attendance and a delay to their surgical treatment. The provision of neoADT for patients during the delay yielded no improvements to peri-operative measures and LOS, when compared to patients not experiencing delays to treatment prior to the Covid-19 pandemic. Given the trend of patients with delayed treatment experiencing increased pN disease, additional neoADT for a subgroup of patients within the covid cohort demonstrated a trend towards pathological downstaging (pT), reduced PSMs and node-positive disease. Treatment delays led to significant rates of detectable PSA. The three-month neoADT duration in this study was inadequate to significantly influence post-operative detectable PSA levels amongst patients in the covid cohort. This data should be treated with caution until longer term biochemical recurrence is studied.
